# Relationship between loneliness and internet addiction: a meta-analysis

**DOI:** 10.1186/s12889-024-18366-4

**Published:** 2024-03-19

**Authors:** Yue Wang, Youlai Zeng

**Affiliations:** Department of Education, Liaoning Normal University, 116000 Dalian, China

**Keywords:** Loneliness, Internet addiction, Meta-analysis

## Abstract

**Background:**

In the digital age, the Internet has become integrated into all aspects of people’s work, study, entertainment, and other activities, leading to a dramatic increase in the frequency of Internet use. However, excessive Internet use has negative effects on the body, psychology, and many other aspects. This study aims to systematically analyze the research findings on the relationship between loneliness and Internet addiction to obtain a more objective, comprehensive effect size.

**Methods:**

This study employed a comprehensive meta-analysis of empirical research conducted over the past two decades to investigate the relationship between loneliness and Internet addiction, with a focus on the moderating variables influencing this relationship. This meta-analysis adopted a unique approach by categorizing moderating variables into two distinct groups: the objective characteristics of research subjects and the subjective characteristics of researchers. It sheds light on the multifaceted factors that influence the relationship between loneliness and Internet addiction.

**Results:**

A literature search in web of science yielded 32 independent effect sizes involving 35,623 subjects. Heterogeneity testing indicated that a random effects model was appropriate. A funnel plot and Begg and Mazumdar’s rank correlation test revealed no publication bias in this meta-analysis. Following the effect size test, it was evident that loneliness was significantly and positively correlated with Internet addiction (*r* = 0.291, *p* < 0.001). The moderating effect analysis showed that objective characteristics significantly affected the relationship. However, subjective characteristics did not affect the relationship.

**Conclusions:**

The study revealed a moderately positive correlation between loneliness and Internet addiction. Moreover, this correlation’s strength was found to be influenced by various factors, including gender, age, grade, and the region of the subjects. However, it was not affected by variables such as the measurement tool, research design, or research year (whether before or after COVID-19).

## Introduction

In the digital age, the Internet has become integrated into all aspects of people’s work, study, entertainment, and other activities, leading to a dramatic increase in the frequency of Internet use. However, excessive Internet use has negative effects on the body (vision, sleep, obesity, sedentary lifestyle, and musculoskeletal disorders) [1], psychology (depression, anxiety, and loneliness), academic performance [2], cognitive ability [3], interpersonal relationships [4], and many other aspects. Kraut, R. et al., were the first to investigate the effects of Internet use on individual social participation and psychological health [5], and since then, the exploration of the relationship between Internet addiction and loneliness has garnered significant attention from scholars.

### The concept of loneliness

In his seminal work, Robert S. stated that loneliness is a subjective psychological feeling or experience in which an individual lacks satisfactory interpersonal relationships due to a gap between their desired social interaction and the actual level [6]. Subsequent research has presented varying definitions of loneliness by different psychologists. Behaviorists believe that loneliness arises from a response to inadequate social reinforcement. Cognitive theorists emphasize that loneliness is a perception resulting from an inconsistency between desired and actual social interactions. Psychoanalytic schools posit that loneliness is related to unfulfilled individual social interaction needs [7].

### The concept of internet addiction

Internet Addiction Disorder (IAD), also known as Internet addiction, was first proposed by Goldberg in 1995. He argued that Internet addiction, as a coping mechanism, is a way of relieving stress and is characterized by excessive Internet use [8]. This concept gained prominence through Young’s pioneering study in 1996. Internet addiction is a problematic behavior defined as an impulse control disorder that does not involve substance addiction. It can have negative effects on academics, relationships, finances, careers, and physical well-being [9].

Scholars have used different theoretical models and terminology to describe excessive Internet use behavior, with the most commonly used terms being “Internet addiction” and “pathological Internet use”. Davis developed a cognitive-behavioral model to explain the causes of pathological Internet use (PIU), emphasizing that individual thoughts play a crucial role in abnormal behavior. Individuals with negative self-perceptions and views of the world receive positive reinforcement through Internet use, which leads to continued and increasingly frequent Internet use. Davis categorized pathological Internet use into two types: specific pathological Internet use, which involves the overuse or misuse of specific Internet functions, and generalized pathological Internet use, which is characterized by pervasive and excessive Internet use, particularly for online socialization [10].

This paper uses the term “Internet addiction” to define excessive Internet use behavior. First, the term “specific pathological Internet use” refers to the overuse of specific online activities, while “generalized pathological Internet use” emphasizes the social function of Internet use. Internet addiction encompasses a wide range of addictive activities and Internet functions, with addiction measured by Internet addiction scales fully reflecting the severity of the issue. Second, the severity of Internet addiction can be expressed on a continuum of problem severity. The term “pathological Internet use” falls in the middle range of problem severity, producing a more benign negative impact. However, “Internet addiction” lies at the top of the continuum and is characterized by more severe consequences [11]. This paper underscores the negative effects of excessive Internet use by using the term “Internet addiction”.

### The relationship between loneliness and internet addiction

In the academic community, three primary research conclusions have emerged regarding the relationship between loneliness and Internet addiction:

#### Loneliness leading to internet addiction

Research indicates that loneliness serves as a predictive factor for Internet addiction [12, 13]. Studies, including one conducted during the COVID−19 pandemic, have consistently shown that loneliness significantly predicts Internet addiction [14]. It is suggested that lonely individuals may resort to excessive Internet use as a coping mechanism to seek emotional support and social interaction [15].

#### Internet addiction leading to loneliness

Another perspective posits that Internet addiction contributes to feelings of loneliness. Research has demonstrated a positive correlation between Internet addiction and loneliness, indicating that individuals with higher levels of Internet addiction tend to experience a stronger sense of loneliness [16]. This is often attributed to the isolation resulting from excessive online engagement, leading to reduced social and family interactions [17].

#### A vicious cycle of loneliness and internet addiction

The third perspective suggests that loneliness and Internet addiction interact in a reinforcing cycle. Studies have shown that lonely individuals are more likely to exhibit Internet addiction behaviors, which, in turn, exacerbate their loneliness [18]. Conversely, excessive Internet use can intensify feelings of loneliness, creating a vicious cycle [19]. Scholars have confirmed the existence of a clear and strong bidirectional relationship between Internet addiction and loneliness [20]. However, this bidirectional relationship is complexity; using the Internet to replace offline social interaction can increase loneliness, while using it to enhance or expand social connections may reduce loneliness [21].

These three perspectives provide valuable insights into the intricate relationship between loneliness and Internet addiction, shedding light on the various pathways through which these phenomena interact.

### The moderating variables of the relationship between loneliness and internet addiction

#### Gender

Research findings on the gender effects of Internet addiction vary widely. Some studies confirm that the prevalence of Internet addiction is significantly higher in women than in men (male = 24%, female = 48%) [22]. Conversely, there are contrary conclusions suggesting that Internet addiction is more common among men [23–25]. However, some studies have shown that there is no significant gender difference in Internet addiction [26].

Similarly, there is no consensus on the gender effect of loneliness in research. Women have higher rates of loneliness than men (male = 23.3%, female = 28.3%) and are more likely to feel a lack of companionship [27]. On the other hand, some studies have shown that loneliness is more common in males than in females [28].

Research on the relationship between loneliness and Internet addiction found no gender differences [29, 30]. However, the results of another meta-analysis showed that, as a moderating variable, the association between Internet addiction and loneliness among females was weak [31]. Therefore, we propose the first hypothesis that there may be a moderating effect of gender (male and female) on the relationship between loneliness and Internet addiction.

#### Age

Current research on the age effect of Internet addiction has not yielded consistent conclusions. Numerous studies have shown that younger Internet users are more prone to Internet addiction than older users [32, 33]. Teenagers who feel lonely are more likely to alleviate their depression and stress through the Internet, leading to Internet addiction [34]. There are also studies showing that both middle-aged and elderly people are inclined to excessive Internet use [35].

Similarly, studies on the age effect of loneliness have not been consistent. Loneliness is not only common phenomenon among adults, with a high prevalence among those aged 60 and above (20–30%) [36], but also among adolescents under 25 (5–10%) [37, 38].

Research has shown that there is no statistically significant difference between adolescents and adults in the effect sizes of the relationship between loneliness and Internet addiction [39]. Similar studies have found no differences in the relationship among children, adolescents, college students, adults, and the elderly [30]. To further investigate whether age has a moderating effect on the relationship, this study proposes the second hypothesis that there is a moderating effect of age (adolescent and adult) on the relationship between loneliness and Internet addiction.

#### Grade

Current research on the grade effect of Internet addiction has not yielded consistent conclusions. Few studies have examined the relationship across different grades, including primary schools, secondary schools, and universities. Some studies found no significant difference in the severity of Internet addiction among these grades [40]. In contrast, other studies have reported significant differences in Internet addiction rates across different grades [23]. Research conducted in middle schools suggests that as grades increase, the rate of Internet addiction gradually rises [41]. For instance, eighth-grade students have been found to be more addicted to the Internet than sixth-grade students (6th graders = 36.7%, 8th graders = 24%) [42]. Furthermore, students in secondary schools tend to show higher levels of Internet addiction than those in middle schools [43]. Among college students, Internet addiction tends to increase with the progression of the school year (1st graders = 8.4%, 2nd graders = 11.5%, 3rd graders = 11.1%, 4th or 5th graders = 12.9%) [23]. Some studies have reported similar conclusions, with a higher prevalence rate of Internet addiction as grade level increases [44]. However, there are also studies that have reached opposite conclusions [45].

Currently, research on the role of grade in regulating loneliness has not reached a consensus. Changes in the level of loneliness among middle school students have not been statistically significant [46, 47]. However, in college, the level of loneliness in freshmen is significantly higher than that in other grades [48].

Research on the relationship between loneliness and Internet addiction has shown a statistically significant and highly positive correlation among middle school students of different grades [49]. Nevertheless, some scholars have found that there is no difference in the relationship between the two regarding grades [31]. In light of these varying findings, this study proposes the third research hypothesis, suggesting that grade (primary schools, secondary schools, and university) has a moderating effect on the relationship between loneliness and Internet addiction.

#### Region

Current research on the regional effects of Internet addiction has not reached a consistent conclusion. Studies have shown that in comparison to Asia and Europe, the severity of Internet addiction in Oceania (Australia and New Zealand) is lower [50]. However, one study found that the Italian sample had the highest mean value of Internet addiction, while the Chinese sample had the lowest mean value of Internet addiction [51].

Similarly, research on the regional effects of loneliness has failed to yield consistent conclusions. The loneliness of teenagers is lowest in Southeast Asia and highest in the eastern Mediterranean region. Among adults, middle-aged individuals, and elderly individuals, the sense of loneliness is lowest in Northern countries and highest in Eastern European countries (Northern European countries = 2.9%, 1.8–4.5%, Eastern European countries = 7.5%, 5.9–9.4% ) [52].

Research has shown that regions have a moderating effect on the relationship between loneliness and Internet addiction, with the correlation between loneliness and Internet addiction in non-Chinese cultures being significantly higher than that in Chinese backgrounds [39]. Therefore, to further explore regional differences, we propose the fourth research hypothesis that region [East Asia (China), West Asia (Turkey, Kuwait, and Saudi Arabia), South Asia (India, Bangladesh), Southeast Asia (Thailand, Malaysia), and Europe (Greece)] has a moderating effect on the relationship between loneliness and Internet addiction.

#### Measurement tool

Russell, an early advocate of the one-dimensional structure of loneliness, argued that there is no difference in the core nature of loneliness, and all lonely individuals understand and experience loneliness in the same way. Consequently, he developed the first edition (1978) of the UCLA (University of California at Los Angeles) Loneliness Scale, which comprised 20 items and had a reliability coefficient of 0.96 [53]. However, because all the items pointed to loneliness, respondents may provide a single response, potentially leading to result deviation. The second edition (1980) of the UCLA Loneliness Scale addressed this issue by including 10 positive and 10 negative items, with the negatively scored items converted to calculate the total score alongside the other items. A higher total score indicates a stronger sense of loneliness, and the reliability coefficient of the scale is 0.94 [54]. Early studies primarily focused on college students with high reading ability. As research deepened, Russell’s third edition (1996) of the UCLA Loneliness Scale underwent simplification and became applicable to various groups. The scale now includes 11 positive items and 9 negative items, rated using a 4-point Likert scale. Its reliability coefficient ranges from 0.89 to 0.94 [55]. The UCLA Loneliness Scale has been adapted into Chinese by Wang, D [56]., Turkish by Demir, A. G [57]., Thai by Wongpakaran, T. et al. [58], and various other versions. Additionally, the Children’s Loneliness Scale, developed by Asher, S. R. et al. is a multidimensional scale containing 24 items designed to measure children’s subjective feelings of loneliness in grades 3–6. Sixteen main items assess loneliness, while eight supplemental items inquire about children’s hobbies and activity preferences, allowing children to answer more honestly and relaxedly. The scale is rated on a 5-point Likert scale with a reliability coefficient of 0.90 for the main items [59]. The Chinese Children’s Loneliness Scale was translated by Wang and other scholars [60] and adapted by Li, X. et al. for middle school students [61].

Young (1996) developed the first Internet addiction screening tool, Young’s Diagnostic Questionnaire for Internet addiction (YDQ), based on the diagnostic criteria for pathological gambling in the Diagnostic and Statistical Manual of Mental Disorders-Fourth Edition (DSM-IV). YDQ is a self-report checklist consisting of 8 yes/no screening criteria, with a diagnosis of Internet addiction requiring the satisfaction of five criteria [62]. In subsequent studies, Young (1998) expanded the scale to 12 items and renamed it the Internet Addiction Test (IAT), which uses a Likert-5 scale with 20 items to measure the presence and severity of Internet addiction [63]. Respondents can be classified as normal, mild, moderate, or severe Internet addicts based on their scores [64]. The IAT is the most widely used scale to measure Internet addiction, gaining international recognition for its reliability and consistency [65]. It has been translated into multiple national versions, including Chinese [66], French [67], Italian [68], Turkish [69], Greek [70], Thai [71], Finnish [72], Korean [73], and Malay [74]. Additionally, the Chinese scholars Chen, S.H. et al. developed the Revised Chen Internet Addiction Scale (CIAS-R), which includes 26 items rated on a Likert-4 scale to assess Internet addiction [75]. It covers core symptoms and related problems of Internet addiction, with dimensions consistent with Block’s proposal of four dimensions involved in Internet addiction [76]. The CIAS-R has been validated by a large number of studies in Taiwan and mainland China and has been adapted into a Turkish version [77].

Differences exist in the dimensions, diagnostic criteria, and focus of measurement tools established on the basis of various theoretical models [78]. Meta-analysis has revealed significant variations in the measurement of Internet addiction when different tools are employed [79]. Studies have shown that the prevalence rates of Internet addiction measured by different measurement tools, were YDQ-8, YDQ-10, IAT and CIAS in increasing order (8.4%, 9.3%, 11.2%, 14.0%, respectively) [23]. It has also been observed that scores measured by the IAT have the highest correlation with loneliness. This may be because the IAT places greater emphasis on evaluating the symptoms [80].

Furthermore, another study confirmed the moderating effect of the Internet addiction measurement tool on the relationship between loneliness and Internet addiction [39]. In light of these findings, this study proposes the fifth research hypothesis that the measurement tools (YDQ, IAT, and CIAS) have a moderating effect on the relationship between loneliness and Internet addiction.

#### Research design

In a cross-sectional study design, data collection occurs at a specific point in time. In contrast, a longitudinal study design involves data collection at predetermined time intervals or fixed events, with subjects continuously tracked over time. Research has demonstrated that compared to cross-sectional studies, longitudinal designs offer a unique perspective on preventing loneliness [81].

Therefore, this meta-analysis introduces the sixth research hypothesis: the study design (cross-sectional study and longitudinal study) has a moderating effect on the relationship between loneliness and Internet addiction.

#### Research year

Research has revealed that with the increase in Internet usage time, Internet addiction has become a prominent issue during the COVID-19 [82]. Scholars have compared people’s levels of loneliness before and after the pandemic. Longitudinal studies have shown that loneliness levels increased after the pandemic [83]. As most reports have noted, people often feel lonely during COVID-19 [84]. However, there are also studies that have reached the opposite conclusion [85].

Statistical analysis indicates that before COVID-19, during the early stage and the recovery stage of the pandemic, the level of Internet addiction among groups with more severe Internet addiction has declined [86]. This meta-analysis proposes the seventh research hypothesis: that the research year (before and after COVID-19) has a moderating effect on the relationship between loneliness and Internet addiction.

Due to differences in research subjects, research tools [49] and measurement methods, there are inconsistencies and even contradictions in research conclusions. For example, scholars point out that the two variables are positively correlated (*r* = 0.43) [87], while Turan, N. et al. have concluded that there is a negative correlation between them (*r*=-0.154) [88]. Using meta-analysis, this study aims to systematically analyze the research findings on the relationship between loneliness and Internet addiction to obtain a more objective, comprehensive effect size. Simultaneously, it seeks to investigate the moderating effects of the objective characteristics of research subjects (gender, age, grade, and region) and the subjective characteristics of researchers (measurement tools, research design, and research year whether before or after COVID-19) on the relationship between loneliness and Internet addiction, with the intention of providing references for subsequent studies.

## Methods

### Eligibility criteria

Population, Intervention, Comparison(s) and Outcome (PICO) is usually used for systematic review and meta-analysis of clinical trial study. For the study without Intervention or Comparison(s), it is enough to use P (Population) and O (Outcome) only to formulate a research question [89]. A well-formulated question creates the structure and delineates the approach to defining research objectives [90].

#### Population

Studies involved both Internet addictive and non-Internet addictive samples. Research is only limited to Internet addiction, not to social media addiction, digital game addiction or smartphone addiction. We did not have any exclusion criteria regarding demographic (gender, age, grade, region) or the research design and research year of the study.

#### Outcome

The outcome was the correlation coefficient of relationship between loneliness and Internet addiction. Regarding the measurement of variables, the inclusive articles use the generally recognized and report the adequate information on reliability and consistency of measurement tools. We include articles using Children’s Loneliness Scale, UCLA Loneliness Scale to measure the level of loneliness and YDQ, IAT, or CIAS to measure Internet addiction.

#### Literature selection criteria

First, we collected empirical studies on the relationship between loneliness and Internet addiction, excluding theoretical studies or review articles. Second, we selected studies that employed quantitative empirical research methods with complete and explicit data. These studies reported correlation coefficients or statistics (e.g., F values, t values, or χ2 values) that could be transformed into correlation coefficients. Third, the literature had to explicitly report the measurement tools used for assessing loneliness and Internet addiction. Fourth, we excluded duplicate publications and included only one instance of repeated data.

### Search strategy

**Fig. 1:**
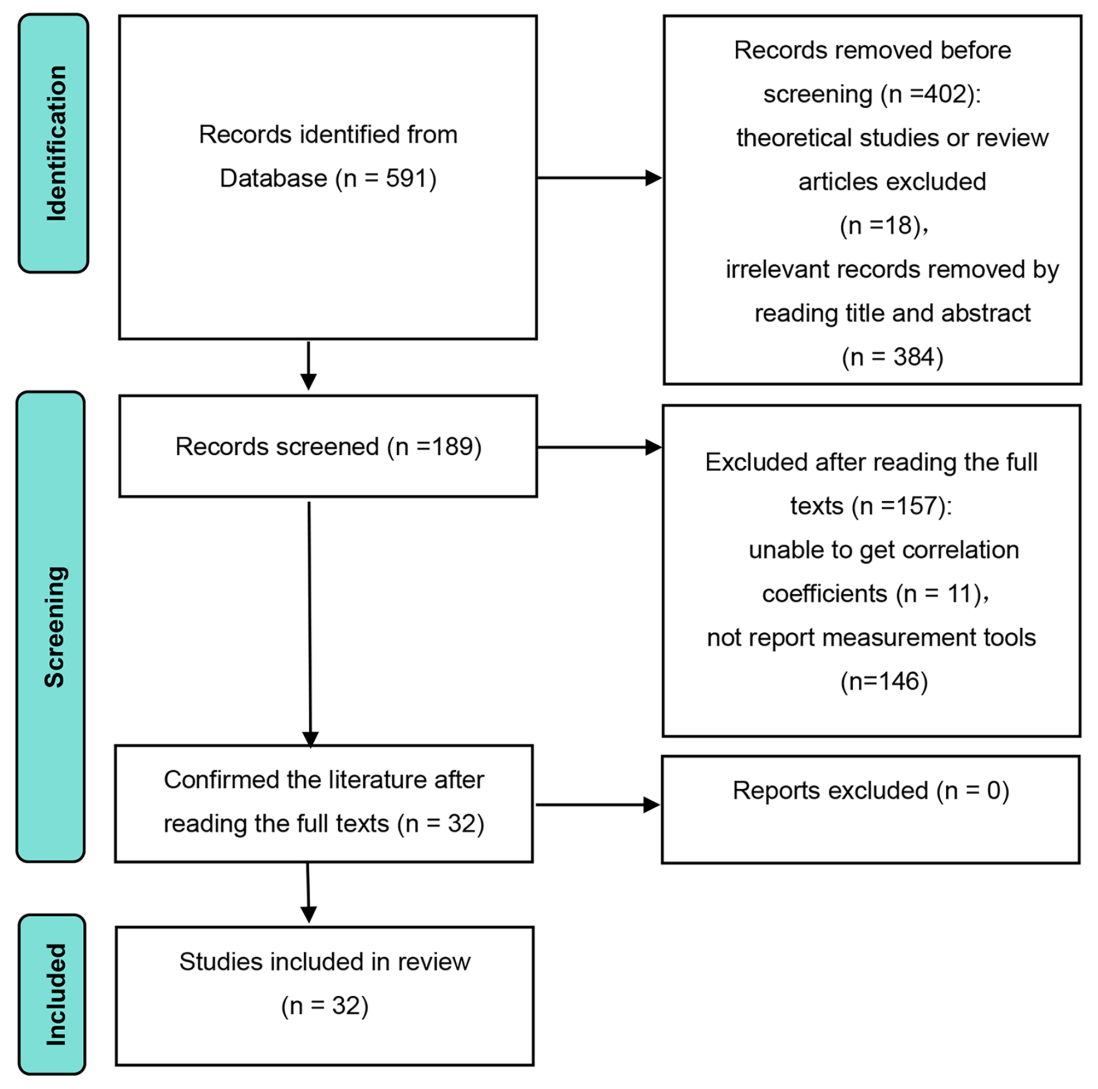
The PRISMA flow chart used to identify studies for detailed analysis of loneliness and Internet addiction

The literature search was divided into three steps. In the first step, we initiated the retrieval process. Internet addiction was formally proposed in 1996, and the literature search included articles published from 1996. The search was conducted in Web of Science using the keywords “Internet addiction” and “loneliness”. The deadline for the literature search was June 25, 2023. Based on our research topic, we initially collected 591 articles. In the second step, we conducted screening and removed an additional 157 articles that did not meet the screening criteria. In the third step, we confirmed the inclusion of 32 articles for meta-analysis after reading the full texts again. In total, the final set of literature included in the meta-analysis consisted of 32 articles, encompassing 32 effect sizes. The flow chart of the literature selection process is depicted in Fig. 1.

### Document coding

The articles included in the meta-analysis were coded using the following categories: (a) references (independent or first author, and year), (b) sample, (c) correlation coefficient, (d) gender (percentage of males), (e) age (adolescent and adult), (f) grade (primary schools, secondary schools, and university), (g) region [East Asia (China), West Asia (Turkey, Kuwait, Saudi Arabia), South Asia (India, Bangladesh), Southeast Asia (Thailand, Malaysia), and Europe (Greece)], (h) measurement tool (YDQ, IAT-12, IAT-20, and CIAS), (i) research design (cross-sectional study and longitudinal study) and (j) research year (before and after the COVID-19 pandemic). The final coding results of 32 target articles were shown in Table 1.

**Table 1:** Basic information of the original study included in the analysis

References	*N*	r	Male%	Age	Grade	Region	Measurement tool of IA	Research design	Research year
Alheneidi, H.(2021) [87]	593	0.43	32	adult	university	West Asia	IAT-20	cross-sectional study	after
Andreou, E.(2013) [91]	384	0.16	45.6	adolescent	secondary schools	Europe	IAT-20	cross-sectional study	before
Bakioglu, F.(2020) [92]	325	0.61	42.2	adult	university	West Asia	IAT-12	cross-sectional study	before
Bozoglan, B.(2013) [93]	384	0.605	29.7	adult	university	West Asia	CIAS	cross-sectional study	before
Cao, Q.(2020) [94]	1218	0.241	55.25	adolescent	primary schools	East Asia	YDQ	cross-sectional study	before
Cheung, C.S.(2018) [95]	665	0.191	51.7	adolescent	secondary schools	East Asia	CIAS	cross-sectional study	before
Eldeleklioglu, J.(2013) [96]	206	0.17	44.2	adolescent	secondary schools	West Asia	IAT-20	cross-sectional study	before
Hong, M.(2021) [97]	364	0.44	33.5	adult	university	East Asia	IAT-20	cross-sectional study	before
Karakose, T.(2022) [14]	432	0.149	42.1	adult	secondary schools	West Asia	IAT-12	cross-sectional study	after
Koyuncu, T.(2014) [98]	1157	0.121	55	adolescent	secondary schools	West Asia	IAT-20	cross-sectional study	before
Li, W.(2016) [99]	73	0.544	53.4	adult	university	East Asia	CIAS	longitudinal study	before
Lin, X.(2018) [100]	626	0.34	41.5	adult	university	East Asia	IAT-20	cross-sectional study	before
Mamun, M.A.(2020) [101]	605	0.188	51.6	adult	university	South Asia	IAT-20	cross-sectional study	before
Ozdemir, Y.(2014) [102]	648	0.32	66	adult	university	West Asia	IAT-20	cross-sectional study	before
Oztekin, C.(2020) [103]	203	0.277	0	adult	university	West Asia	IAT-12	longitudinal study	before
Ozturk, A.(2021) [104]	1028	0.525	39.7	adult	university	West Asia	IAT-20	cross-sectional study	before
Peng,C.(2021) [105]	15,232	0.26	51.8	adolescent	secondary schools	East Asia	IAT-20	cross-sectional study	before
Senormanci,O.(2014) [106]	40	0.045	100	adult	university	West Asia	IAT-20	cross-sectional study	before
Shi, X.(2017) [29]	3289	0.221	41.3	adolescent	secondary schools	East Asia	YDQ	cross-sectional study	before
Shi, X.(2023) [107]	3363	0.22	45.6	adolescent	secondary schools	East Asia	YDQ	cross-sectional study	after
Simcharoen, S.(2018) [108]	324	0.292	43.2	adult	university	Southeast Asia	IAT-20	cross-sectional study	before
Tan, K.A.(2019) [109]	207	0.21	30	adult	university	Southeast Asia	YDQ	cross-sectional study	before
Tian, Y.(2020) [110]	1047	0.285	44.85	adolescent	secondary schools	East Asia	CIAS	longitudinal study	before
Turan, N.(2020) [88]	160	−0.154	6.9	adult	university	West Asia	IAT-20	cross-sectional study	before
Wongpakaran, N.(2021) [111]	318	0.319	43	adult	university	Southeast Asia	IAT-20	cross-sectional study	before
Yang, Y.(2022) [112]	241	0.209	51	adult	secondary schools	East Asia	CIAS	cross-sectional study	after
Yang, H.(2022) [35]	446	0.152	48.9	adult	university	East Asia	CIAS	cross-sectional study	after
Yao, M.Z.(2014) [1]	361	0.36	51.7	adult	university	East Asia	IAT-20	cross-sectional study	before
Zeng, W.(2016) [113]	624	0.29	49.7	adolescent	secondary schools	East Asia	IAT-20	cross-sectional study	before
Zhang, S.(2018) [114]	169	0.295	47.9	adolescent	university	East Asia	CIAS	cross-sectional study	before
Zhao, Y.(2022) [115]	783	0.35	50.3	adult	university	East Asia	CIAS	cross-sectional study	after
Zhao, Y.(2022) [116]	108	0.3	63	adolescent	university	East Asia	CIAS	cross-sectional study	after

### Data analysis

In this study, we employed Comprehensive Meta Analysis 3.0 (CMA 3.0) for our meta-analysis. The effect size used for analysis was the correlation coefficient. To combine the effect sizes from the included studies, we chose the random effects model for statistical models that account for the potential variability between studies.

The random effects model assumes that each study is drawn from different aggregates, leading to significant variability among studies. As we aimed to investigate the moderating effects of various variables, these differences among studies could influence the final results. Therefore, the use of the random effects model was appropriate for evaluating the effect sizes. The results are measured by the effect sizes. Below 0.2 is low level effect, 0.2–0.5 is moderate low level, 0.5–0.8 is upper medium level, and above 0.8 is high effect level [117]. The heterogeneity between studies was tested with Higgins’ criteria for I^2^, values of 25%, 50%, and 75% correspond to low, moderate, and high degrees of heterogeneity, respectively [118].

## Results

### Sample characteristics

This meta-analysis incorporated data from 32 independent samples, encompassing a total of 35,623 subjects. The age coverage of the study population is wide, the grades are concentrated in senior grades, like secondary schools and university. Subjects on the relationship between Internet addiction and loneliness are mostly located in Asian countries. IAT-20 is the most used questionnaire to measure Internet addiction, and the CIAS is mostly used by Chinese scholars. The research design was mostly cross-sectional study, and the research year were evenly distributed in the period of 2013–2023.

### Homogeneity test

In the heterogeneity test, the results in Table 2 indicated significant heterogeneity (Q = 395.797, I^2^ = 92.168, *p* < 0.001). This finding suggests that a substantial proportion, 92.168%, of the observed variance in the relationship between loneliness and Internet addiction is attributed to real differences in this relationship. Additionally, the Tau-squared value was 0.013, indicating that 1.3% of the variation between studies could be considered for the calculation of the weights.

Given the high heterogeneity observed, a random effects model was appropriately employed for the meta-analysis. This aligns with the inference that the relationship between loneliness and Internet addiction is influenced by certain moderating variables.

**Table 2:** Results of the heterogeneity test for the effect sizes of loneliness and Internet addiction

Model	Number Studies	SMD	95% interval		Heterogeneity			
			Lower limit	Upper limit	χ^2^	df	*p*	I^2^
FEM	32	0.269	0.259	0.278	395.797	31	0.000	92.168%
REM	32	0.291	0.251	0.331				

### Assessment of publication bias

**Fig. 2:**
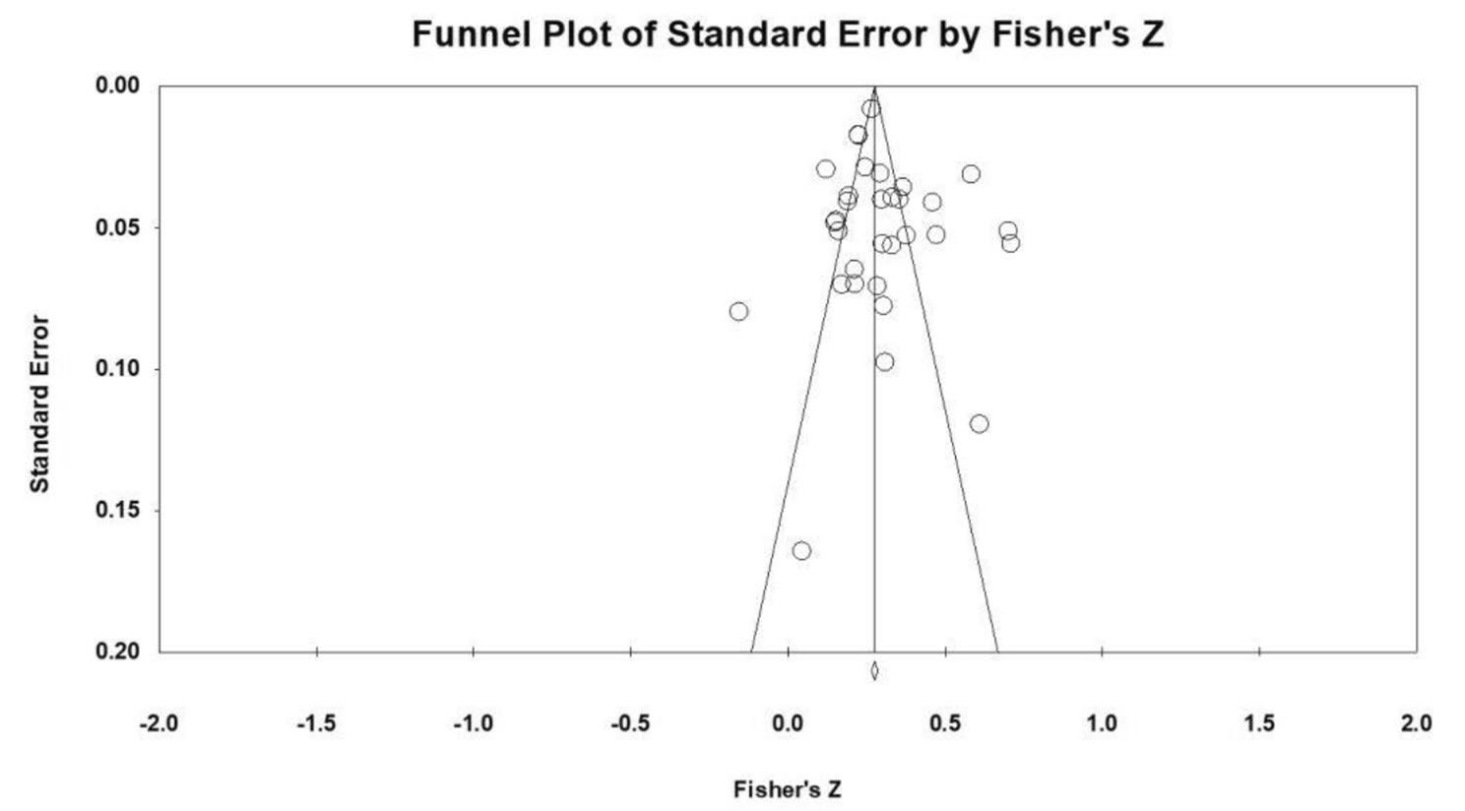
Funnel plot of effect sizes of the correlation between loneliness and Internet addiction

As evident from Fig. 2, the literature included in the meta-analysis was distributed on both sides of the center line. Notably, there are relatively few points on the bottom-right side of the funnel plot, indicating a small number of studies with large effect sizes and potentially low accuracy. Conversely, the majority of points cluster at the top of the funnel plot, suggesting small errors and large sample sizes.

These observations collectively indicate that meta-analysis is minimally affected by publication bias. The distribution of studies and the symmetry of the funnel plot suggest that the included literature provides a balanced representation of the relationship between loneliness and Internet addiction.

To further objectively evaluate publication bias, we conducted Begg and Mazumdar’s rank correlation test. The results showed that Kendall’s Tau was 0.06855 (*p* > 0.05), indicating that there was no evidence of publication bias in the meta-analysis. These findings align with the observations from the funnel plot, reaffirming the absence of publication bias in the study.

### Main effect test

**Fig. 3:**
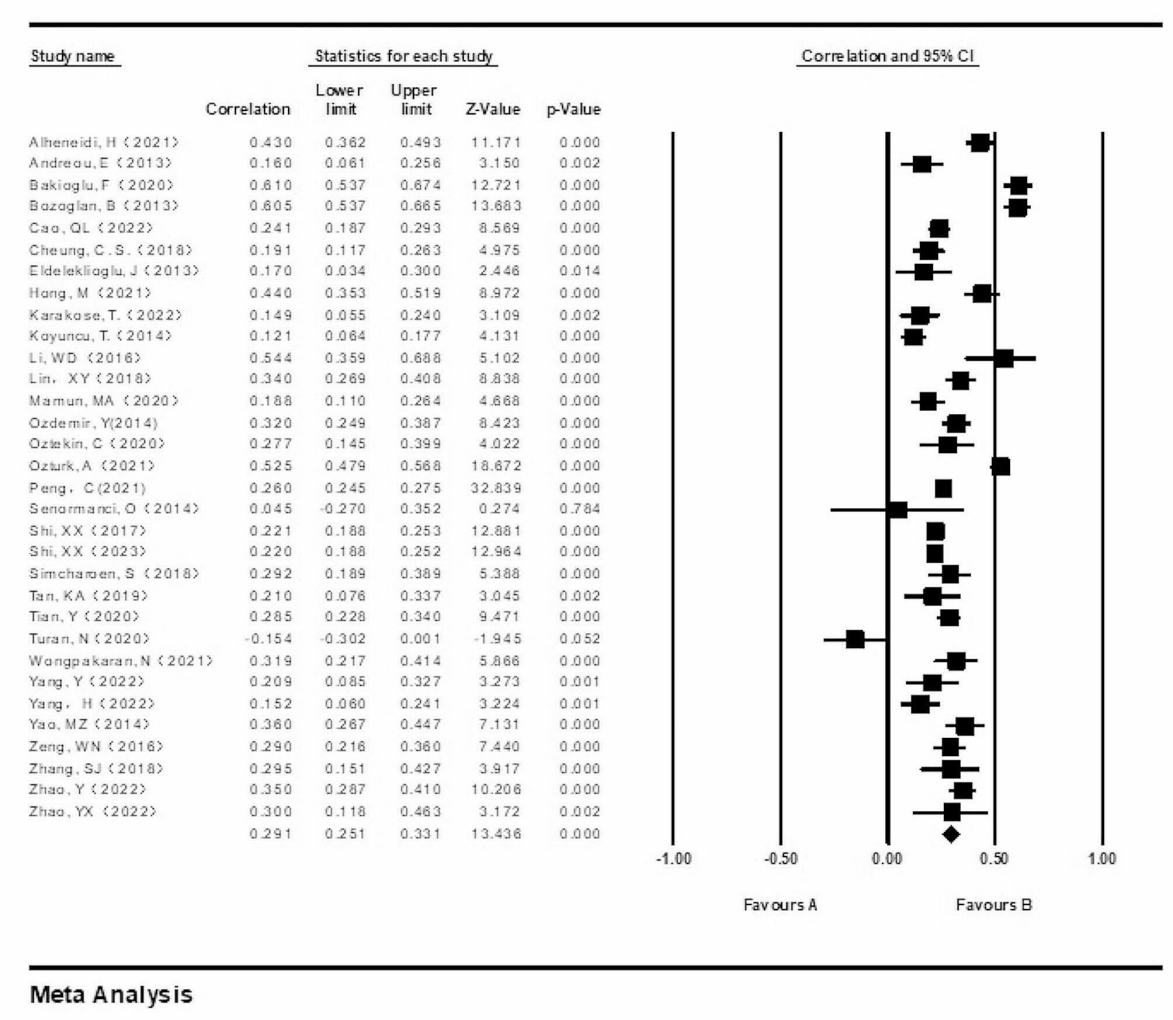
Forest plot of the comprehensive effects of loneliness and Internet addiction

We employed a random effects model to assess the main effects of the eligible literature, the results were shown in Fig. 3. The results from the random effects model revealed a correlation coefficient of 0.291 (95% CI = 0.251–0.331, Z = 13.436, *p* < 0.001). This finding suggests a moderately positive correlation between loneliness and Internet addiction.

### Moderating effect test

This study investigated the moderating impact of both objective characteristics of subjects and subjective characteristics of researchers on the relationship between loneliness and Internet addiction, and the findings are summarized in Table 3. The results revealed that several subject characteristics—gender (Qb = 4.159, *p* < 0.05), age (Qb = 5.879, *p* < 0.05), grade (Qb = 9.281, *p* < 0.05), and region (Qb = 9.787, *p* < 0.05)—influenced the association between loneliness and Internet addiction. Specifically, as the proportion of males increased, the correlation coefficient between Internet addiction and loneliness was significantly lower than that observed among females. Moreover, the correlation between loneliness and Internet addiction was notably lower in adolescents than that in adults. Furthermore, the strength of the relationship was significantly lower among primary and secondary school students than that among university students. Additionally, region-specific variations emerged, indicating that the correlation between loneliness and Internet addiction increased sequentially in Europe, South Asia, East Asia, Southeast Asia, and West Asia.

However, we found no significant moderating effects related to the measurement tool (Qb = 6.573, *P* > 0.05), research design (Qb = 0.672, *P* > 0.05), or research year relative to COVID-19 (Qb = 0.633, *P* > 0.05) on the relationship between loneliness and Internet addiction.

**Table 3:** Moderating effects of the relationship between loneliness and Internet addiction

	Moderator	Category	k	r	95%CI	Qb(df)	*P*
Objective characteristics of subjects	Male%	0-44%	17	0.328	0.248,0.405	4.159(1)	0.041
		45-100%	15	0.237	0.202,0.272		
	Age	adolescent	12	0.227	0.198,0.255	5.879(1)	0.015
		adult	20	0.328	0.252,0.400		
	Grade	primary schools	1	0.240	0.187,0.293	9.281(2)	0.010
		secondary schools	11	0.215	0.183,0.246		
		university	20	0.340	0.266,0.410		
	Region	East Asia	16	0.277	0.247,0.306	9.787(4)	0.044
		West Asia	11	0.309	0.167,0.438		
		South Asia	1	0.188	0.110,0.264		
		Southeast Asia	3	0.283	0.219,0.344		
		Europe	1	0.160	0.061,0.256		
Subjective characteristics of researchers	Measurement tool of IA	YDQ	4	0.223	0.202,0.244	6.573(3)	0.087
		IAT-12	3	0.364	0.027,0.626		
		IAT-20	16	0.277	0.213,0.338		
		CIAS	9	0.328	0.225,0.424		
	Research design	cross-sectional study	29	0.286	0.243,0.328	0.672(1)	0.412
		longitudinal study	3	0.342	0.212,0.460		
	Research year	before	25	0.300	0.250,0.347	0.633(1)	0.426
		after	7	0.262	0.180,0.340		

## Discussion

### Relationship between loneliness and internet addiction

This study conducted a comprehensive meta-analysis of empirical research conducted over the past two decades to examine the relationship between loneliness and Internet addiction. It incorporated data from 32 studies involving a total of 35,623 subjects. The findings confirmed a significant positive correlation between loneliness and Internet addiction (*r* = 0.291, *p* < 0.001), underscoring a moderate relationship between two variables. These results align with the conclusions of previous study [119]. According to problem-behavior theory, problem behavior is defined as behavior that is socially disapproved by the institutions of authority. Problem behavior may be an instrumental effort to attain goals that are blocked or that seem otherwise unattainable [120]. Unmet needs such as loneliness lead them to seek solace in the online world and perpetuating a cycle of loneliness.

Notably, this meta-analysis adopted a unique approach by categorizing moderating variables into two distinct groups: the objective characteristics of research subjects and the subjective characteristics of researchers. It sheds light on the multifaceted factors that influence the relationship between loneliness and Internet addiction. Furthermore, it explored the impact of research design on these findings, providing novel insights into this relationship.

In addition to these contributions, this study also considered global COVID-19, incorporating literature published after the outbreak. This allowed for an investigation into the influence of the pandemic on the relationship between loneliness and Internet addiction. This meta-analysis thus provides a comprehensive understanding of the evolving dynamics between loneliness and Internet addiction.

### Moderating effect of the relationship between loneliness and internet addiction

#### The moderating role of gender

This study categorized the proportion of male participants into two groups and found that as the proportion of male participants increased, the correlation between loneliness and Internet addiction gradually decreased, with statistically significant differences between the groups. These results, contrary to previous findings [31], warrant further investigation.

Analyzing the reasons behind this, it is worth noting that men and women often differ in the functions of Internet use. Women tend to use it for socializing and meeting interpersonal needs, while men are more inclined to spend time on online games to fulfill self-actualization and personal needs [121]. Studies have also shown that women exhibit a stronger correlation between social use of the Internet and loneliness, while men display a stronger correlation between leisure use and loneliness compared to women [122]. Additionally, women may be more vulnerable to Internet addiction [123].

#### The moderating role of age

The study confirmed that loneliness is significantly less associated with Internet addiction in adolescents than in adults. Loneliness is with a high prevalence among adults [124], and the incidence of Internet addiction in adults is also high [50]. Adolescents, who often study and live in collective environments with peer support and parental supervision, are less likely to feel lonely and become addicted to the Internet. In contrast, adults may use the Internet as a means to escape life pressures, leading to increased loneliness due to excessive online engagement.

#### The moderating role of grade

The findings indicated that the correlation between loneliness and Internet addiction is significantly lower among primary and secondary school students than among university students. The results are consistent with the conclusions of the existing studies [45]. Primary school students’ immaturity, limited self-control, and susceptibility to Internet addiction contribute to this pattern. Secondary school students, focused on academic pressures, tend to have the lowest correlation between loneliness and Internet addiction. Conversely, in addition to academic pressure, there are two important tasks for university students: forming identity and building meaningful and intimate relationships. Many people have not achieved an independent identity and remain overly attached to their families. This may cause the sense of loneliness, Internet addiction as one of the coping mechanisms to alleviate psychological problems [125].

#### The moderating role of region

The correlation coefficients between loneliness and Internet addiction varied across regions, with Europe exhibiting a lower correlation compared to Asian regions. The result support a previous cross-national meta-analysis study [126]. Some European countries have implemented policies and regulations to curb Internet addiction, which has had a controlling effect [127]. However, it is essential to note that the European and South Asian subgroups included only one study, potentially affecting the findings.

#### The moderating role of measurement tool

The results suggested that the measurement tool used did not significantly moderate the relationship between loneliness and Internet addiction. This is consistent with the conclusions of the existing studies that even different instruments give comparable results [128]. This underscores the consistency and scientific validity of the measurement tools. However, it is worth exploring the impact of different thresholds within the IAT-20 scale on the relationship between loneliness and Internet addiction in future studies, as there have been discrepancies in threshold selections [129].

#### The moderating role of research design

Interestingly, the research design was found to have no significant moderating effect on the relationship between loneliness and Internet addiction. This suggests that research results are robust across different research designs, even though cross-sectional research designs have been subject to credibility concerns in social science research.

#### The moderating role of research year

The analysis revealed that the research year did not moderate the relationship between loneliness and Internet addiction. This underscores the stability and resilience of this relationship, which is unaffected by external events such as the COVID-19.

### Limitations

In the analysis of moderating effects, the sample distribution of certain moderating variables was not adequately balanced, and the sample sizes for specific subgroups were relatively small. For instance, variables such as grade (primary school) and region (Europe and South Asia) which had only one data point is also included, in order to ensure the integrity and authenticity of the data. This could impact the accuracy of the moderating effects analysis.

## Conclusions

This study employed a meta-analysis methodology and CMA 3.0 (Comprehensive Meta-analysis 3.0) to quantitatively analyze 32 foreign literature sources examining the relationship between loneliness and Internet addiction. The primary objectives were to objectively estimate the overall effect size of loneliness and Internet addiction and to investigate how research characteristics might moderate this effect.

The study’s findings revealed a moderately positive correlation between loneliness and Internet addiction. Moreover, this correlation’s strength was found to be influenced by various factors, including gender, age, grade, and the region of the subjects. However, it was not affected by variables such as the measurement tool, research design, or research year (whether before or after COVID-19).

In summary, this meta-analysis suggests a noticeable link between loneliness and Internet addiction, with specific demographic and contextual factors impacting the strength of this relationship.

## Data Availability

Data can be requested from the corresponding author.

## Abbreviations

CIAS-R: Revised Chen Internet Addiction Scale
DSM-IV: Diagnostic and Statistical Manual of Mental Disorders—Fourth Edition
IAD: Internet Addiction Disorder
IAT: Internet Addiction Test
PICO: Population, Intervention, Comparison(s) and Outcome
PIU: Pathological Internet Use
YDQ: Young’s Diagnostic Questionnaire for Internet addiction
